# Postsynaptic densities fragment into subcomplexes upon sonication

**DOI:** 10.1186/s13041-019-0491-y

**Published:** 2019-08-22

**Authors:** Ayse Dosemeci, Jung-Hwa Tao-Cheng, Valerie Bakly, Thomas S. Reese

**Affiliations:** 1 Laboratory of Neurobiology, National Institute of Neurological Disorders and Stroke, National Institutes of Health, Bethesda, MD USA; 2 EM Facility, National Institute of Neurological Disorders and Stroke, National Institutes of Health, Bethesda, MD USA

**Keywords:** PSD, Postsynaptic density, EM, Electron microscopy, Sonication

## Abstract

Postsynaptic density (PSD) fractions were isolated from rat forebrain and sonicated. Pellets from sonicated samples examined by electron microscopy revealed particles with an electron density similar to PSDs that appeared to be fragments of PSDs. Immuno-gold labeling confirmed that some of these contained PSD-95 and/or SynGAP. Biochemical analysis of supernatant and pellet fractions from sonicated samples showed almost complete recovery of several major PSD components (SynGAP, PSD-95, Shank3, Homer and Glutamate receptors) in the pellet, while the supernatant contained known contaminants of PSD fractions, such as glial acidic fibrillary protein and neurofilament protein, as well as actin and α-actinin, indicating susceptibility of these cytoskeletal elements to mechanical disruption. Size distributions of particulate material in control and sonicated samples were clearly different, with particles in the 40–90 nm range observed only in sonicated samples. Fragmentation of the PSD into subcomplexes containing major constituents suggests a patchwork structure consisting of weakly bound modules, that can be readily dissociated from each other through mechanical disruption. Modular organization and weak association between modules would endow the PSD with lateral structural flexibility.

**Electronic supplementary material:**

The online version of this article (10.1186/s13041-019-0491-y) contains supplementary material, which is available to authorized users.

## Introduction

PSD is a protein complex lining the intracellular side of the postsynaptic membrane in excitatory synapses. The complex is disk-shaped, with average areas ranging from 0.04 to 2.4 μm^2^ for different types of spines [1, 2]. Specialized proteins associate with each other to form a scaffold and other PSD elements such as receptors, auxiliary proteins and enzymes bind to scaffold elements forming a spatially organized network. A pertinent question that remains to be resolved is whether the PSD is organized around a single continuous scaffold encompassing its whole volume, or is it a patchwork of modules, each organized around its separate scaffold?

Certain observations point out the existence of discrete multiprotein complexes of different sizes within the PSD. Studies using super-resolution imaging reveal the presence of ‘nanodomains’ (70–80 nm) or ‘nanoclusters’ (140–170 nm) of PSD-95 and/or AMPA receptors [3–5] and ‘nanocolumns’ [6] and ‘nanomodules’ [7] spanning pre- and postsynaptic compartments. Other studies adopted chemical strategies to isolate and characterize complexes of PSD proteins. Treatment of subcellular fractions from brain with ionic detergents at pH 8/9 extracts complexes of different sizes and compositions. These include recent studies using Blue Native PAGE that identified 1.5 MDa complexes, containing PSD-95 and receptors or other proteins [8].

In the present study we adopted a new approach: mechanical disruption of PSD preparations by sonication to separate PSD subcomplexes. Our assumption in choosing sonication was that weak associations would be especially susceptible to mechanical disruption. In addition, we expected that mechanical treatment would act on a large range of protein-protein interactions rather than targeting specific types (polar, hydrophobic, etc.). We report fragmentation of the PSD into subcomplexes through sonication suggesting a modular structure.

## Methods

### Antibodies

(Protein: Company (Catalogue #) host/type dilution for Western)

SynGAPα1: Millipore (06–900) rabbit polyclonal 1:1000; PSD-95: New England Peptide (custom) rabbit polyclonal 1:1000; Shank3: Santa Cruz (30193) rabbit polyclonal 1:50; Homer: Synaptic Systems (160103) rabbit polyclonal 1:500; Actin: Chemicon (MAB1501R) mouse monoclonal 1:100; α-actinin: Millipore (MAB1682) mouse monoclonal 1:100; IRSp53: NeuroMAB (L117/1) mouse monoclonal 1:4; NF-L: Sigma (N5139) mouse monoclonal 1:200; GFAP: Sigma (G3893) mouse monoclonal 1:2000; GluA1: Synaptic Systems (182003) rabbit polyclonal 1:500; GluA2: Millipore (MAB397) mouse monoclonal 1:500; GluN2A: Upstate (06–313) rabbit polyclonal 1:1000; GluN2B: NeuroMAB (N59/20) mouse monoclonal 1:125.

For immuno-electron microscopy: SynGAPα2: Abcam (EPR2883Y) rabbit monoclonal 1:200; PSD-95: New England Peptide (custom) rabbit polyclonal 1:200.

### Preparation of PSD fraction

Brains from 7 to 12 week-old rats of both gender were custom collected and immediately frozen in liquid nitrogen by Rockland (Gilbertsville, PA). PSD fractions from cerebral cortices were prepared as described previously [9]. Briefly, a synaptosome fraction was treated with 0.5% TritonX-100. The detergent-insoluble pellet was further fractionated by sucrose density centrifugation and a crude PSD fraction was collected from the 1.5 M/2.1 M sucrose interface. After a second extraction with TritonX-100/75 mM KCl, the PSD fraction was collected over a 2.1 M sucrose cushion.

### Sonication and separation of particulate material by centrifugation

A probe sonicator, Kontes KT50 micro ultrasonic cell disruptor (frequency 20KHz), was used for sonication. PSD preparation (250 μg protein) was resuspended in 2.5 ml of 20 mM HEPES pH 7 and was sonicated at an output amplitude setting of 40%, in a tube placed on ice, four times for 20s each, with 40 s cooling intervals. An aliquot (1 ml) was taken out and labeled as ‘sonicated’. The rest (1.5 ml) was further sonicated at an output amplitude setting of 100%, two times for 20s each, with a 100 s cooling interval and labeled as ‘sonicated+’. An unsonicated control sample 100 μg/ml was set aside in ice. Control, ‘sonicated’ and ‘sonicated+’ samples, each containing 100 μg protein in 1 ml, were centrifuged in a refrigerated bench centrifuge with a swinging bucket rotor at 11,700 g for 100 min at 4 °C. Supernatants were removed and pellets were either fixed in 4% glutaraldehyde in preparation for electron microscopy or solubilized in SDS-containing sample buffer in preparation for electrophoresis. Protein in supernatants was recovered by precipitation with TCA and re-solubilized in SDS-containing sample buffer before electrophoresis. For immunogold labelling, samples, 250 μg/ml, were sonicated as above, and 600 μl aliquots were centrifuged at 50,000 rpm (~ 237,000 g) for 60 min at 4 °C in a Beckman SW55 rotor.

### Electrophoresis and Western immunoblotting

4–15% Mini-PROTEAN TGX Precast polyacrylamide gels (BioRad) were used for SDS-PAGE. Gels were transferred to PVDF membranes using the Trans-Blot Turbo Transfer System (BioRad), blocked, incubated with primary and secondary antibodies, and visualized via chemiluminescence.

### Electron microscopy

Samples for structural analysis were fixed with 4% glutaraldehyde in 0.1 M cacodylate buffer at pH 7.4 at room temperature for 30 min and stored at 4 °C. Fixed pellets were processed in the centrifuge tubes until the embedding step. Samples were washed in buffer and treated with 1% osmium tetroxide in buffer for 1 h on ice, washed and treated with 1% uranyl acetate in acetate buffer at pH 5.0 at 4 °C overnight. Samples were then washed and dehydrated through a graded series of ethanol and embedded in epoxy resins. Prior to embedding, pellets were removed from the centrifuge tubes, trimmed and oriented so that they were sectioned vertically through the thickest portion of the pellets. In order to document the gradient of components within the pellets, photographs were taken consecutively from top to bottom of the pellets. Since the smaller fragments were concentrated at the top of the pellets, sampling for length measurements was restricted to the top 30 μm of the pellets. Every PSD and PSD fragment photographed within this top portion of the pellets was measured for statistical analysis. To estimate the size of PSD and PSD fragments, the length (major axis) of these structures were measured and plotted into histograms. The criteria for sampling a “PSD fragment” for measurement was similarity of electron density to the parent control PSDs. The smallest entity that could be unequivocally defined as a “PSD fragment” was ~ 40 nm in length.

Samples for immunogold labeling were fixed with 4% paraformaldehyde in PBS at room temperature for 20–30 min, washed in PBS and stored at 4 °C. Fixed pellets were removed from the centrifuge tubes and cut into 4 quadrants so that the cut surfaces could be exposed to immunolabeling reagents. All immunolabeling steps were carried out at room temperature. Samples were treated with 5% normal goat serum and 0.1% saponin in PBS for 30 min, incubated with primary antibody for 1 h, washed with PBS and then incubated with secondary antibody conjugated with a small gold (Nanogold at 1:200, Nanoprobes, Yaphand, NY) for 1 h, washed and fixed with 2% glutaraldehyde in PBS and stored at 4 °C. Controls for immunolabeling included omitting primary antibodies or using other primary antibodies in the same immunolabeling runs. Samples were then washed in deionized water and silver enhanced (HQ kit from Nanoprobes) to enlarge the small gold to visible sizes, treated with 0.2% osmium tetroxide on ice for 30 min, followed by 0.25% uranyl acetate at 4 °C for 30 min, dehydrated through a graded series of ethanol and embedded in epoxy resins. Samples were oriented so that they were sectioned through the thickest portion of the pellets. Since immunoreagents only penetrate ~ 10 μm into the tightly pelleted sample, only the cut surfaces of the pellets were examined.

Sections of 70–90 nm thickness were counterstained with uranyl acetate and lead citrate. Images were photographed with a bottom-mounted digital CCD camera (AMT XR-100, Danvers, MA, USA).

## Results

**Fig. 1:**
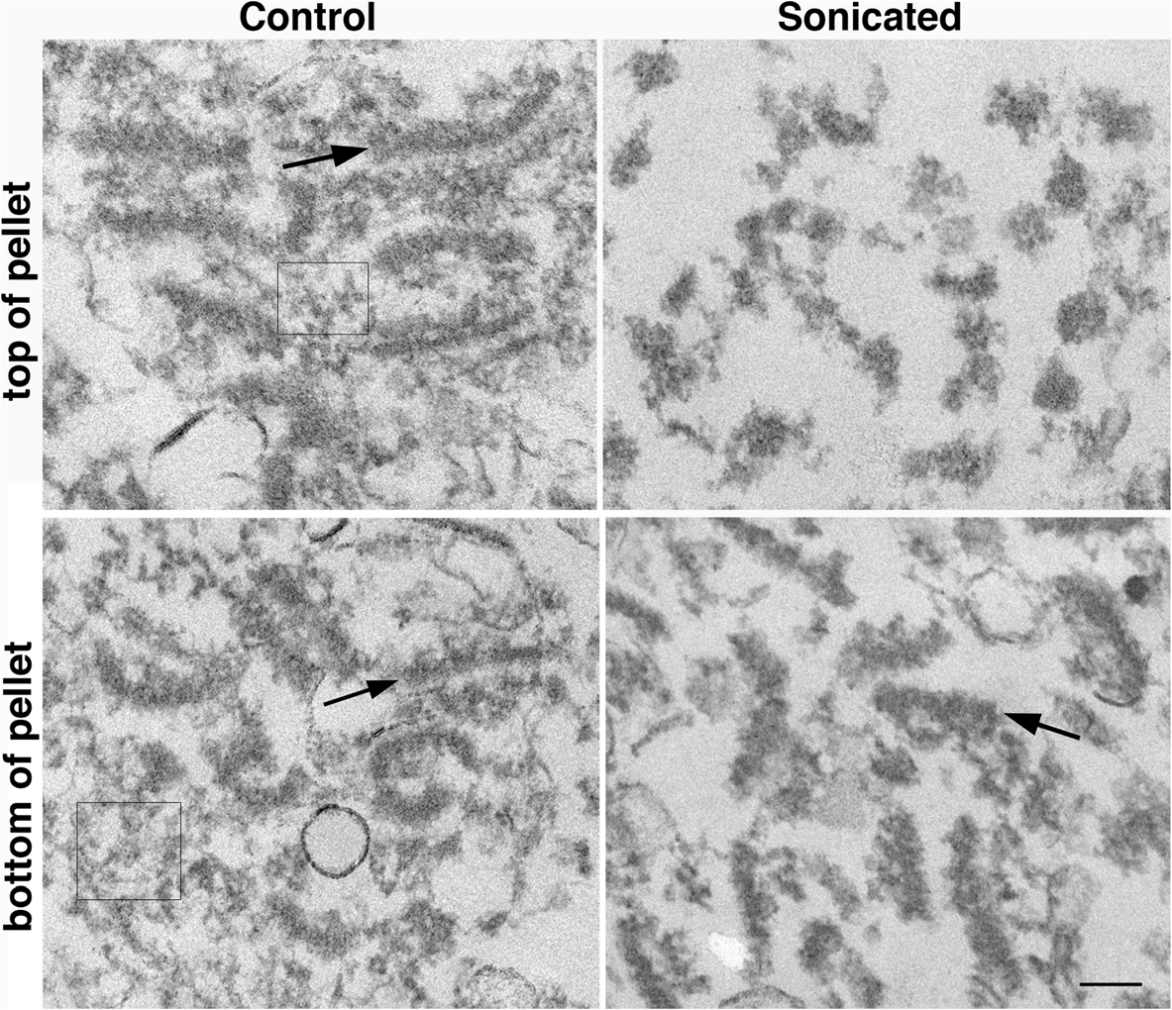
Particles that may be fragments of PSDs are produced upon sonication. PSD fractions resuspended in 20 mM HEPES were sonicated with a probe sonicator as described in Methods; unsonicated control (left panels) and sonicated (right panels) samples were centrifuged. Pellets were fixed and processed for electron microscopy. Sections covering the entire depth of the pellets from top (top panels) to bottom (bottom panels) were examined by electron microscopy. Arrows point to typical PSDs, and boxes (left panels) depict areas containing probable cytoskeletal contaminants. Scale bar: 100 nm

**Fig. 2:**
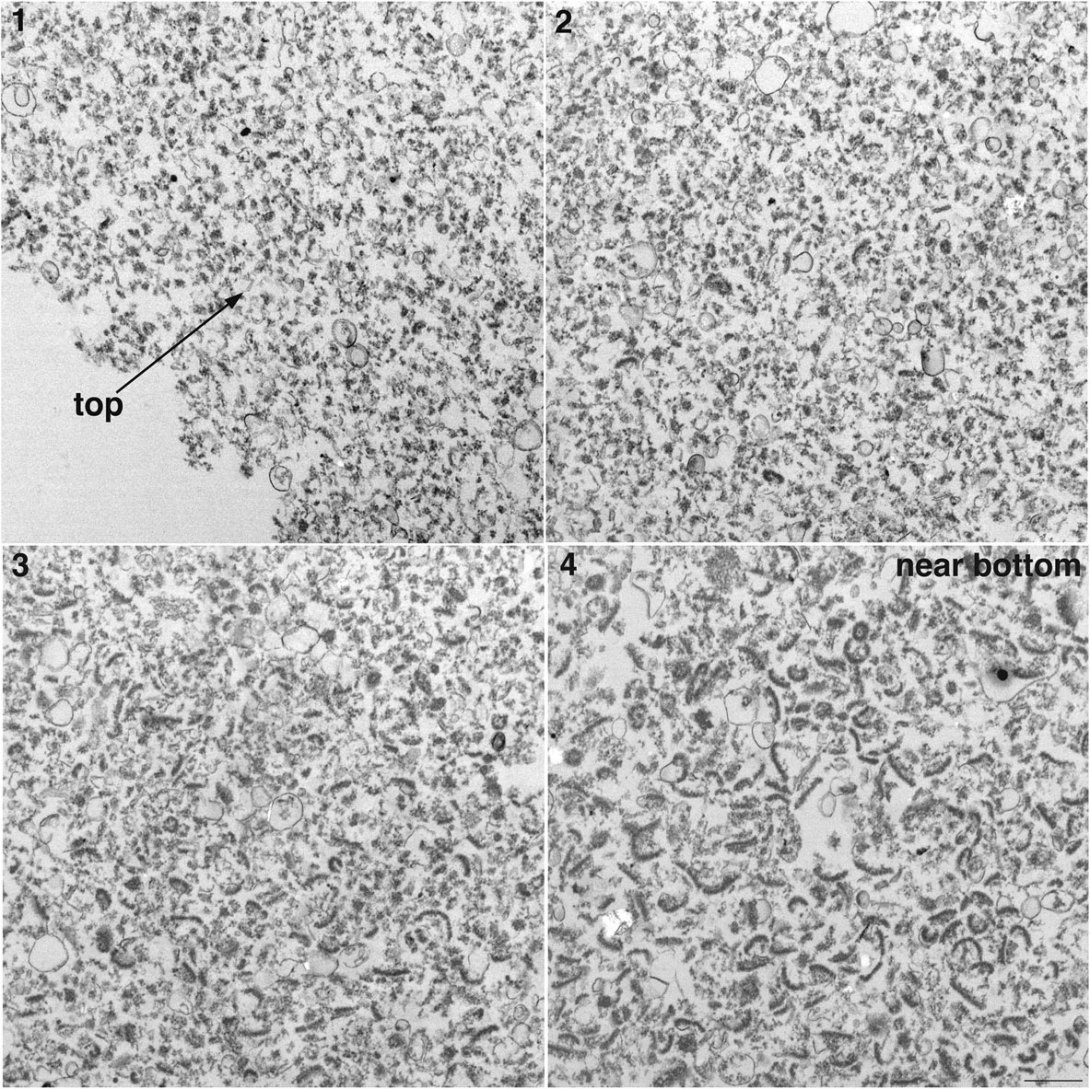
Electron micrographs at low magnification show particle size gradient in pellets from sonicated samples: EM images (1–4) were taken at ~ 70 nm intervals from the top (1) to near bottom (4) of the pellet from a sonicated sample. Scale bar: 500 nm

PSD preparations were sonicated (4 X 20s, output amplitude setting of 40%) and centrifuged. Pelleted particulate material from control and sonicated samples was examined by electron microscopy. In control samples, material with the characteristic morphology of PSDs was observed within the entire depth of the pellets from top to bottom (Fig. 1, left panels). In sonicated samples, particulate material distinctly smaller than PSDs was observed in the top portions of the pellet (Fig. 1, upper right; Fig. 2, panel 1). Going from top to bottom of the pellet, an increasing proportion of typical PSDs were observed together with smaller particulate material (Fig. 2), with mostly PSDs at the very bottom (Fig. 1, lower right; Fig. 2, panel 4). The smaller particulate material observed in sonicated samples had a similar electron density to PSDs, suggesting that they are fragments of the PSD. On the other hand, fuzzy material in control samples (Fig. 1, boxed areas) which may be contaminating cytoskeletal elements [10] was greatly reduced in pellets from sonicated samples. This difference between control and sonicated samples persisted throughout the different layers of the pellets. In the remaining of text, we shall refer to PSDs and smaller particulate material of similar electron density as ‘particles’.

**Fig. 3:**
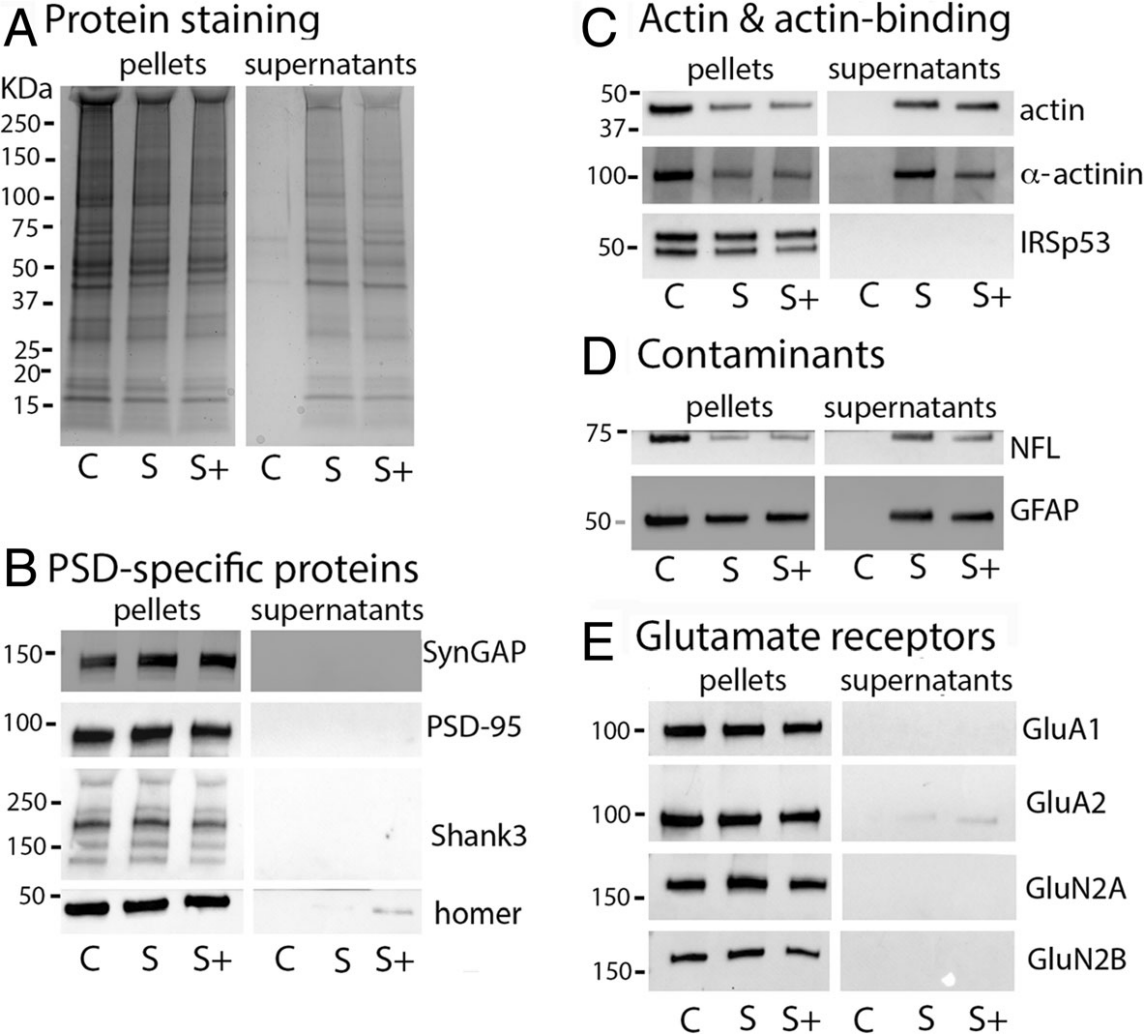
Sonication promotes dissociation of cytoskeletal elements (c&d) while major PSD constituents (b&e) are recovered in the pellet. Three types of samples, control (C), sonicated (S) and sonicated+ (S+, additional sonication at output amplitude setting of 100%), were prepared and centrifuged as detailed in Methods. Supernatants and pellets corresponding to 10 μg original protein were separated by SDS-PAGE and Western immunoblots were obtained using indicated antibodies

Examination of pellets by electron microscopy suggests that sonication breaks the PSD into fragments. In order to test whether sonication also causes dissociation of some proteins from the PSD, supernatants and pellets from control and sonicated samples were analyzed by electrophoresis and Western immunoblotting. Figure 3 compares partitioning of proteins in control and sonicated samples into pellets and supernatants. Comparison of Coomassie blue protein-staining profiles of supernatants in control (C) and sonicated (S, S+) samples indicated partitioning of a substantial amount of selected proteins into the supernatant upon sonication (Fig. 3a). Western immunoblotting identified some of the proteins released into the supernatant as actin and α-actinin, two proteins present near/at the PSD and known to interact with PSD components (Fig. 3c). In addition, two known cytoskeletal contaminants in PSD preparations, neurofilament light chain (NF-L) and glial acidic fibrillary protein (GFAP) were recovered in substantial amounts in supernatants (Fig. 3d). On the other hand, none of the PSD-specific proteins tested, as well as none of the glutamate receptor subunits of either AMPA or NMDA type showed appreciable partitioning into the supernatant in sonicated samples (Fig. 3b&e). Additional sonication at output amplitude setting of 100% (S+) appeared to promote a small increase in the release of Homer and of AMPA-type glutamate receptors (Fig. 3b&e). However, the amounts of AMPA receptor subunits and Homer released in the supernatant upon sonication varied among experiments and were smaller when purer PSD preparations (as defined by EM) were used. This suggests that contaminating extrasynaptic elements may be the source of AMPA receptor and Homer released into the supernatant.

**Fig. 4:**
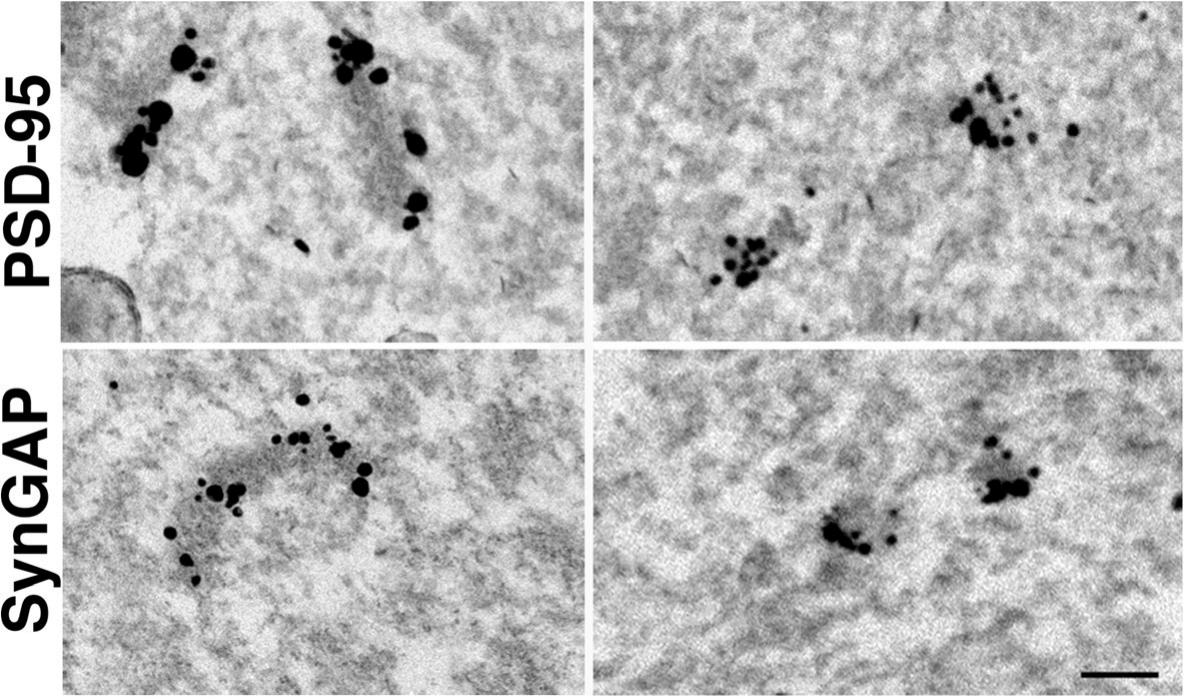
Particles in sonicated samples label for PSD-specific proteins. Pellets from sonicated samples were labeled with antibodies for PSD-95 and SynGAPα2. Electron micrographs show specific labeling of some PSDs (left column) and small particles (right column). Black grains of heterogeneous sizes represent silver-enhanced gold label. Scale bar: 100 nm

Biochemical analysis indicates that, following sonication, the bulk of known PSD components does not dissociate but remains in particulate form. These observations are compatible with the idea that smaller particulate material formed through sonication are fragments of PSDs. Immunogold electron microscopy was conducted to confirm the presence of the two most abundant PSD proteins, PSD-95 and SynGAP, in these particles. With the protocol used, labeling intensity of PSDs and smaller particles was heterogeneous and the labeling efficiency was low (Additional file 1) making quantitative analysis problematic. On the other hand, some particles (small particles as well as PSDs) were clearly labelled indicating the presence of PSD-95 and SynGAP (Fig. 4).

**Fig. 5:**
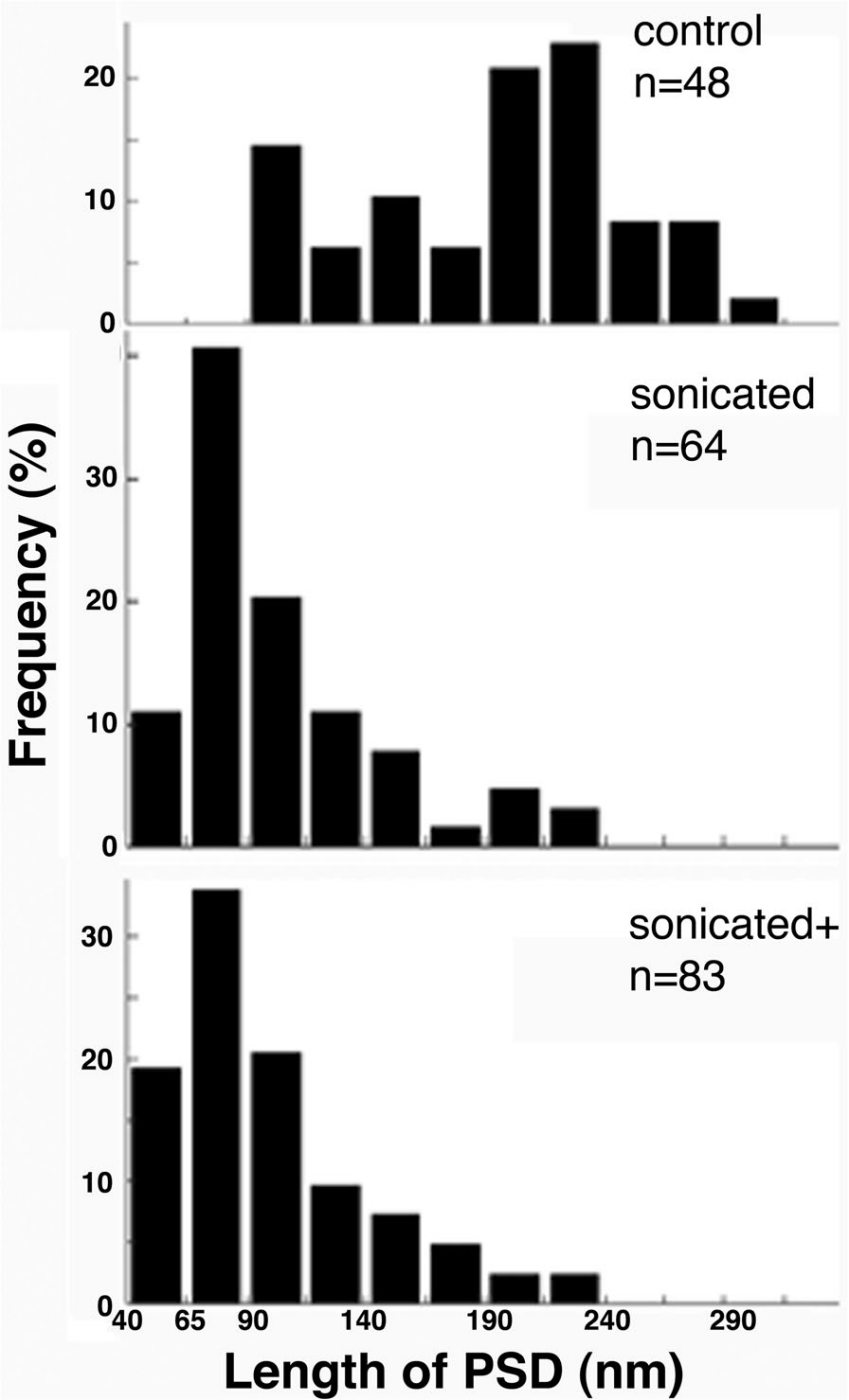
Size distribution of particles in control and sonicated samples. An area within 30 μm from the top of each pellet was sampled and the length (major axis) of PSDs and particles, ~ 40 nm in length and bigger, of similar electron density were measured in electron micrographs from control, sonicated and sonicated + samples. The differences between the control vs. either sonicated or sonicated + samples were statistically significant (*P* < 0.0001 via ANOVA for means, and Wicoxon test for medians) whereas no statistically significant differences were found between sonicated vs sonicated+ samples using the same statistics

A quantitative comparison of particle sizes in control and sonicated samples was carried out to provide further evidence of fragmentation. Comparison of histograms for size distribution shows a clear leftward shift of the distribution in sonicated samples, indicating fragmentation. Most notable is the appearance of particles within 40–90 nm range in sonicated samples (Fig. 5). Since these particles were observed in sonicated samples but not in controls, it can be assumed that they are fragments of PSDs produced through sonication. Additional sonication at maximum output amplitude setting (sonicated+) did not promote a statistically significant change in the mean and median particle size, although a small increase in the percentage of particles in the 40–65 nm range could be observed (Fig. 5). Serial EM confirmed that these small particles were confined within a single thin section, and stereo viewing of tilted images indicates an irregular ball-like shape.

## Discussion

Here we describe a strategy for the fragmentation of PSDs by mechanical means, without the use of chemical reagents. We present electron microscopic and biochemical evidence indicating production of subcomplexes containing major PSD elements upon sonication of PSD preparations.

The PSD preparation contains, in addition to PSDs, contaminating cytoskeletal filaments that are not attached to PSDs [10]. Following centrifugation of sonicated samples, sizable amounts of contaminating GFAP and NF-L as well as actin are recovered in the supernatants, implying depolymerization of these filaments. Consistent with this interpretation, comparison of electron micrographs of pellets from control and sonicated samples indicate disappearance of fuzzy cytoskeletal material upon sonication. Thus, sonication followed by centrifugation can be a useful purification step for the isolation of PSD preparations for biochemical analyses.

The cytoskeleton in dendritic spines is actin-based and PSDs lining the synapse are thought to associate with the actin cytoskeleton through actin-binding proteins [11]. Of the two actin binding proteins prominent in PSD preparations, IRSp53 does not dissociate into the supernatant upon sonication, whereas a major fraction of α-actinin is recovered in the supernatant. IRSp53 is a protein containing multiple protein-protein interaction, demonstrated to be present at the PSD by immuno electron microscopy [12], and shows a stoichiometric ratio of one IRSp53 (BAIP2) to four PSD-95 in PSD preparations [13]. Present results establish IRSp53 as part of PSD subcomplexes and a prime candidate as a linker (adaptor) between the PSD and the actin cytoskeleton.

While mechanical perturbation through sonication is not expected to target a particular type of protein-protein interaction, it can be assumed that the weakest reversible associations would be selectively disrupted. Sonication produces PSD fragments of varying sizes ranging from 40 to 90 nm in length. The smaller size group of particles in the 40–65 nm range may represent discrete protein complexes or ‘modules’ within the PSD. A 3D EM analysis of the structure of these fragments might help understand the overall architecture of the PSD, but was beyond the scope of our present study. Notably, our biochemistry data indicate that the bulk of most PSD proteins remain in the pellets of both sonicated and sonicated + samples, and our EM data show no statistically significant differences in fragment size distribution between sonicated and sonicated + samples, suggesting that PSDs consist of mechanically robust modules.

Knowledge of their size allows a rough estimation of the possible number of subcomplexes or modules that can fit into an average PSD. Assuming an average diameter of 50 nm for the smaller group of subcomplexes (modules), a compact single layer arrangement of modules and a square shape for the PSD, an average PSD with a side length of 360 nm can accommodate ~ 50 average modules. Since the mass of an average PSD was estimated to be ~ 1.1 GDa [14], an average module would have a mass of ~ 0.02 GDa. The estimated mass implies that these complexes can contain multiple copies of PSD scaffolds and receptors as well as multiple copies of 1.5–2 MDa ‘supercomplexes’ as defined by Grant’s group [8]. However, it should be noted that, although the smallest particles that could reasonably be identified as PSD fragments were ~ 40 nm in length, our results do not eliminate the possibility that PSDs could be further broken into pieces smaller than 40 nm.

The size of our ‘modules’ separated by sonication are relatively small compared to nanodomains (70–80 nm, [3, 4]) or nanoclusters (140–170 nm, [5]) described in super-resolution imaging studies. It is conceivable that nanodomains or nanoclusters of PSD-95 and/or AMPA receptors observed in live neurons are composed of two or more ‘modules’ as defined in this study. In this case, reversible association/dissociation and movement of modules could underlie some of the observed time-dependent changes in nanodomains [3].

In conclusion, the pattern of fragmentation of the PSD upon sonication implies a modular organization of the structure. It can be envisaged that subcomplexes, or PSD modules, associate laterally with each other to form a disk underneath the postsynaptic membrane. Modular organization of the PSD may allow lateral movement of subcomplexes and thus regulate aspects of synaptic organization such as apposition of receptors vis-à-vis presynaptic release sites.

## Additional file

Additional file 1: Immunogold labeling of sonicated samples for PSD-95 and SynGAP (PDF 3353 kb)

## Data Availability

The datasets generated and/or analyzed during the current study are available from the corresponding author on reasonable request.
