# Supplementary material for: Postsynaptic densities fragment into subcomplexes upon sonication
Source: Mol Brain. 2019 Aug 22;12:72. doi: 10.1186/s13041-019-0491-y (PMC6704671; doi:10.1186/s13041-019-0491-y)
Supplement: Supplementary file 1 — Immunogold labeling of sonicated samples for PSD-95 and SynGAP (PDF 3353 kb) [file 13041_2019_491_MOESM1_ESM.pdf]

Additional File 1. Immunogold labeling of sonicated samples for PSD-95 and SynGAP

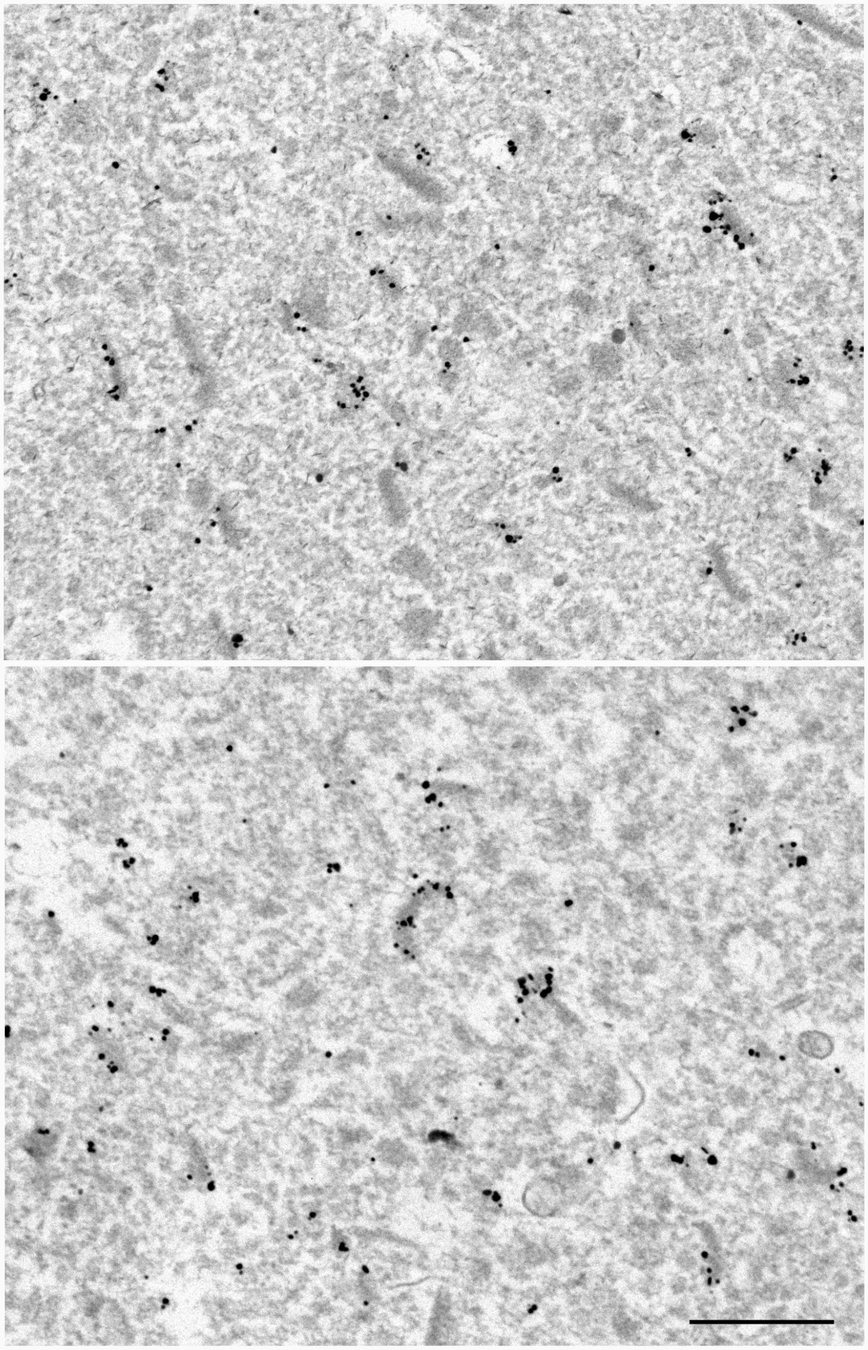

Images were taken from the middle of pellets with PSD fragments of different sizes. Labeling intensity is heterogeneous. Scale bar = 0.5 μm.
